# Cost-effectiveness and budget impact analysis of Daratumumab, Lenalidomide and dexamethasone for relapsed-refractory multiple myeloma

**DOI:** 10.1186/s12962-024-00525-4

**Published:** 2024-02-28

**Authors:** Zahra Goudarzi, Rahil Sadat Shahtaheri, Zhila Najafpour, Haleh Hamedifar, Hamidreza Ebrahimi

**Affiliations:** 1 Health Human Resources Research Center, Department of Health Economics, School of Health Management and Information Sciences, Shiraz University of Medical Sciences, Shiraz, Iran; 2 Department of Pharmacoeconomics and Pharmaceutical Administration, Tehran University of Medical Sciences, Tehran, Iran; 3 Department of Health Care Management, School of Public Health, Ahvaz Jundishapur University of Medical Sciences, Ahvaz, Iran; 4 CinnaGen Medical Biotechnology Research Center, Alborz University of Medical Sciences, Karaj, Iran

**Keywords:** Daratumumab, Carfilzumib, Cost effectiveness analysis, Multiple myeloma

## Abstract

**Background:**

The prominent efficacy in terms of increasing progression-free survival (PFS) of Daratumumab, Lenalidomide and dexamethasone (DRd) triplet therapy versus Carfilzomib, Lenalidomide and dexamethasone (KRd) was proven previously in relapsed-refractory multiple myeloma (RRMM). However, the cost effectiveness of DRd versus KRd is unknown.

**Methods:**

We developed a Markov model by using an Iranian payer perspective and a 10-year time horizon to estimate the healthcare cost, Quality-adjusted life years (QALYs) and life years gain (LYG) for DRd and KRd triplet therapies. Clinical data were obtained from meta-analyses and randomized clinical trials (RCTs). One-way and probabilistic sensitivity analysis were performed to assess model uncertainty. Budget impact analysis of 5 years of treatment under the DRd triplet therapy was also analysed.

**Results:**

DRd was estimated to be more effective compared to KRd, providing 0.28 QALY gain over the modelled horizon. DRd-treated patients incurred $264 in total additional costs. The incremental cost utility ratio (ICUR) and cost effectiveness ratio (ICER) were $956/QALY and $472/LYG respectively.

The budget impact analysis indicates that adding Daratumumab to Lenalidomide and dexamethasone regimen, in the first 5 years, will increase the healthcare system’s expenses by $6.170.582.

**Conclusion:**

DRd triplet therapy compared to KRd is a cost-effective regimen for RRMM under Iran willingness-to-pay threshold.

## Introduction

MM is the second most common haematological malignancy and accounts for approximately 1.8% of all new cancer cases worldwide [1–3]. Myeloma is usually symptomatic and reduces the patient's health-related quality of life (HRQoL) [4]. Most patients also experience comorbidities such as skeletal problems and kidney and heart failure, which significantly increase the burden of the disease [5, 6]. Although the survival rate of patients with myeloma has increased in recent decades (5-year survival probability from 29.2% in 1992 to 57.9% in 2018), most patients with MM after first-line treatment experience relapse, which has increased the economic burden of managing this disease, and in a situation where most health care decision-makers are faced with a lack of resources, the value of drugs for treating MM has received increasing attention [3, 7–9].

The therapeutic goal of treating MM is to achieve the longest PFS with minimal treatment-related toxicity, thus prolonging OS (overall survival), maintaining or improving HRQoL. RRMM is usually treated with a combination of two or three drug classes.

Given the multitude of therapies now approved for the treatment of RRMM, treatment decisions are becoming increasingly complex.

In the past decade, Lenalidomide and/or Bortezomib in combination with dexamethasone have been the most common treatment options for the management of RRMM [10].

Currently, the most common treatment options for RRMM patients who have received at least one treatment in Iran is Carfilzomib in combination with Lenalidomide and dexamethasone.

Carfilzomib is a protease inhibitor that has been approved for use in patients with RRMM in combination with Lenalidomide and dexamethasone (KRd; ASPIRE study) [11] or dexamethasone alone (Kd; ENDEAVOR study) [12–14]. The results of studies show that the addition of Carfilzomib to common treatments significantly increases PFS and OS in patients with RRMM [15–18].

Daratumumab is first fully human monoclonal antibody that has been approved in many countries for the monotherapy treatment of patients with MM [19, 20]. Combined with therapies including Bortezomib/dexamethasone (Vd; CASTOR trial) [21] and Lenalidomide/dexamethasone (Rd; POLLUX trial) [22], this drug significantly prolonged PFS and OS in patients with RRMM. This has led to the approval of combination therapy with Daratumumab in many countries in patients who have previously received more than one treatment line [23].

So far, no study has done a head-to-head comparison of DRd and KRd drug regimens, but a network meta-analysis on RCTs related to RRMM treatments with an emphasis on efficacy measures showed that DRd may currently be the most effective regimen in the RRMM patient [24]. And triple-drug regimens containing Daratumumab, Ixazomib, Carfilzomib, or Elotumumab plus Lenalidomide and dexamethasone can be recommended as first-line treatments for RRMM patients [24].

In contrast to the significant effectiveness of triple-drug regimens, the very high cost of these regimens for long-term treatment is an issue that is considered during clinical decisions and health insurance policy decisions. Despite conducting numerous economic evaluations in patients with RRMM, no study has been conducted to investigate the cost effectiveness of KRd and DRd regimens. Considering that the mentioned treatments are the most common treatment options for RRMM in Iran, the aim of this study is to evaluate the cost-effectiveness of treatment with DRd or KRd from the perspective of Iranian health care payers in patients who have received at least one previous treatment.

## Methods

### Model overview and structure

**Fig. 1:**
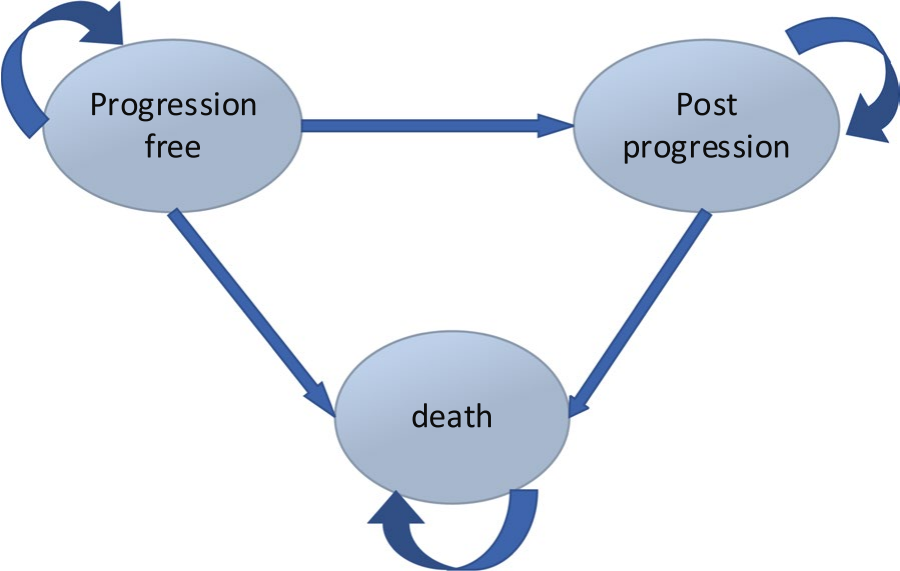
Diagram of cost-effectiveness model

We developed a Markov model to analyse the economic outcomes of DRd in the treatment of RRMM, using 1-month time cycles (Fig. 1).

A literature search was conducted to identify the best available evidence to inform the model structure and parametric input. Time spent in each health state was multiplied by weights for quality of life and direct health care costs from the perspective of an Iranian payer, then accumulated over a lifetime to obtain quality-adjusted life years (QALYs) and total costs. An annual discount rate of 3% and 7.2% was applied for outcomes and costs, respectively [25].

Three health states were considered PFS, post-progression disease (PD), and Death. All patients were categorized in the PFS phase at first. They could then move to the other two states: PD and Death. Patients in the PD state could either remain there or move to the death state. Patients in both PD and PFS could enter the death state. The developed model had several assumptions. Patients follow their treatment choice during the study time horizon and the rate of treatment discontinuations is not included in the model. If the patients go through the treatment period in both arms and the disease does not progress, the Daratumumab and Carfilzumab regimens will not be used and the patients will receive supportive care treatments. As the disease progresses, patients continue to receive treatment.

### Model inputs

#### Data source

Patients characteristics and clinical input parameters (OS, PFS) used in the model were derived from a network meta-analysis that have compared efficacy of treatments for previously treated RRMM [26].

### Patients and interventions

Adults with RRMM disease who had received one to three prior treatments were eligible. Patients who had received Bortezomib treatments previous to the study were also qualified as long as their treatment was progression-free. Patients who had received Lenalidomide and dexamethasone were also qualified as long as their treatment had not stopped due to side effects or their disease had progressed during the first 3 months of treatment. The planned treatment period for the patients who received Daratumumab was 25 months [27]. The planned treatment period for the patients who received Carfilzomib was 18 months [11]. The patients of the Daratumumab group received 400 mg per kilogram of body weight once weekly during treatment cycles 1 and 2, every 2 weeks during cycles 3 through 6, and every 4 weeks thereafter. The Carfilzomib group received 60 mg on days 1, 2, 8, 9, 15, 16 during cycles 1 through 12 and on days 1, 2, 15, 16 during cycles 13 through 18. 40 mg of dexamethasone were prescribed on days 1, 8, 15, 22 during the cycle and 25 mg of Lenalidomide were prescribed on days 1 through 21 in each cycle along with the treatment. According to the network meta-analysis [26], patients treated with DRd showed lower risk of progression or death than KRd with (HR = 0.60, 0.43, 0.82). Also, overall survival of DRd treatment were reported to be better than the survival rates of the KRd treatment (HR = 0.46, 95% CrI = [0.28, 0.75] [24]. The general rate of adverse events (AEs) was extracted [11, 28]and added to the model (Table 1).

**Table 1:** Model input parameters

Parameter	Value	Value lower 95% CI	Value upper 95% CI	Distribution	Source or justification
Mean age of patients	69	NA	NA	Not varied	[27]
Sex of patients (men)	59%	NA	NA	Not varied	[26]
Model cycle length	1 month	NA	NA	Not varied	[11, 28, 29]
Model time horizon	10 years	NA	NA	Not varied	[29]
Probability of AE (adjusted per cycle)					
DRd	0.011	0.009	0.0126	Beta	
KRd	0.026	0.0221	0.0299	Beta	
Cost($)					
Specialist visit	2.08	1.66	2.5	Gamma	Tariff of IranianHS^a^
Labratory tests	20	16	24	Gamma	Tariff of IranianHS
MRI	10.4	8.33	12.5	Gamma	Tariff of IranianHS
CT	11.8	9.4	14.4	Gamma	Tariff of IranianHS
Daratumumab 400 mg	274.2	219.4	329	Gamma	Iranian Food and Drug org
Carfilzomib 60 mg	217	173	260	Gamma	irc.fda.gov.ir/nfi
Dexamethasone 40 mg	0.83	0.66	1	Gamma	irc.fda.gov.ir/nfi
Radiotherapy	14.2	11.4	17	Gamma	Tariff of IranianHS
Lenalidolidomide 25 mg	2.9	2.3	3.5	Gamma	irc.fda.gov.ir/nfi
Drug administration	19.7	15.8	23.6	Gamma	Tariff of IranianHS
Management toxicity	21.6	16.4	25.1	Gamma	Tariff of IranianHS
AE	24.4	19.5	29.2	Gamma	Tariff of IranianHS
Utility					
U_PFS	0.73	0.584	0.876	Betta	[29]
Disutility_AE	− 0.049	− 0.0392	− 0.0588	Betta	[29]
Disutility_Progression	− 0.054	− 0.0432	− 0.0648	Betta	[29]
Discount rate					
Cost	0.072	NA	NA	Not varied	[30]
QALY	0.03	NA	NA	Not varied	

*AE* adverse event, *U* utility, *PFS* progression-free survival, *HS* health system

^a^https://ta.muq.ac.ir

### Medical resource use

We considered the total direct costs of treatment for patients treated with DRd and KRd. The following cost elements were included: drug costs, AE treatment costs (most common AE were diarrhea, fatigue, Cough, Pyrexia, Upper respiratory tract infection, Hypokalemia, management toxicity (include keratopathy, thrombocytopenia, anemia, lymphopenia and neutropenia), drug administration and routine monitoring costs, follow-up treatment, and associated AE costs. All costs are calculated on the 10th of August, 2022 United States dollars (USD) (Table 1).

### Health-state utilities

To measure utility, the QALYs of each health phase were used along with the decrease in utility because of AEs. 1 stands for full health and 0 stands for death. Since the Daratumumab is not used in Iran, we used studies focused on the utility of different MM drugs to extract utility data for our model [29] (Table 1).

## Analysis

In each 1-month cycle, the model generated outcomes that were aggregated to estimate QALYs and lifetime costs for DRd and KRd over a life time horizon. The ICUR and ICER was calculated as incremental costs per QALY gained and incremental costs per LYG gained. An annual discount rate of 3% and 7.2% was applied for outcomes and costs, respectively [25]. All analyses were conducted from an Iranian payer perspective. A willingness to pay (WTP) threshold of 40.000.000 IRR equals to $1290 per QALY gained was used for the analyses.

### Sensitivity analysis

Univariate Deterministic Sensitivity Analysis (DSA) and Probablistic Sensitivity Analysis (PSA) were conducted to test the effects of parameter uncertainty within the model.

For DSA, the model parameters were varied using 95% CIs. If these were not available, ± 20% of the base case values were used (Table 1).

For PSA, Standard probability distributions were assigned to relevant model parameters and 10,000 s-order Monte Carlo simulations were computed.

### Budget impact analysis

Budget impact analysis (BIA) was conducted to estimate the financial budgetary impact of adding Daratumumab to Rd double regimen in the treatment of MM from the payer perspective in Iran. To analyze the impact of the budget according to Iranian guidelines, a 5-year time horizon with total direct medical costs was used. In Iran, MM has an incidence and prevalence rate of 1.8 and 3.27 per 100,000 people, respectively [31]. Since Daratumumab and Carfilzomib are often prescribed for patients who are in the last stage of MM, about 20% of MM patients are transferred to this stage [32]. Based on prevalence and annual incidence rates, we calculated the annual number of eligible Iranian patients at the beginning of the next 5 years. At this stage, KRd is an unrivaled treatment for patients and is therefore assumed to have 100% market share. But with the availability of DRd treatment, carfilzomib's market share is expected to decline in the coming years. Therefore, if access is granted, it is assumed that Daratumumab will capture 5% of market volume in year one, 10% in year two, and finally 25% of market volume in year five. The primary cost drivers for budget impact were medications, diagnostic services, chemotherapy, visits, related adverse events, radiotherapy, and hospitalization. Inflation is not included in the rise in health care costs over the next few years.

## Results

### Base-case results

In the base-case analysis with a life-time horizon, the economic and health outcomes calculated by using the Markov model are presented in Table 2.

**Table 2:** Cost-effectiveness results of base-case scenario

Parameters	DRd group	KRd group	Increment
Cost	15,370	15,106	264
QALY	1.56	1.28	0.28
lYG	5.86	3.52	2.34
ICUR	–	–	956
ICER	–	–	472
ACER	9859	11,773	–
NMB	− 13,359	− 13,451	92

DRd demonstrated better effectiveness compared with KRd with QALYs of 1.56, 95% CI [1.49; 1.62] and 1.28, 95% CI [1.19; 1.37], respectively. Accounting for LYG, patients in DRd group gained 5.86 LYG, 95% CI [5.34; 6.27]; this was 2.34 LYG more than for patients in KRd group. Projected total life-time cost was $15,370, 95% CI [12,616; 18,124] and $15,106, 95% CI [12,231; 17,981] for DRd and KRd regimen. Compared with KRd regimen, the ICUR and ICER for DRd regimen were $956/QALY, 95% CI [654; 14,124] and $472/LYG, 95% CI [265; 932] respectively. The Net Monetary Benefit (NMB) was negative for both treatment strategies, proving that the efficacy and cost of the two regimens are disproportionate. However, since the KRd regimen is less proportional, the DRd method is less expensive. ACER (average cost effectiveness ratio) results show reaching 1 QALY in DRd requires less funds (Table 2).

### Sensitivity analysis

**Fig. 2:**
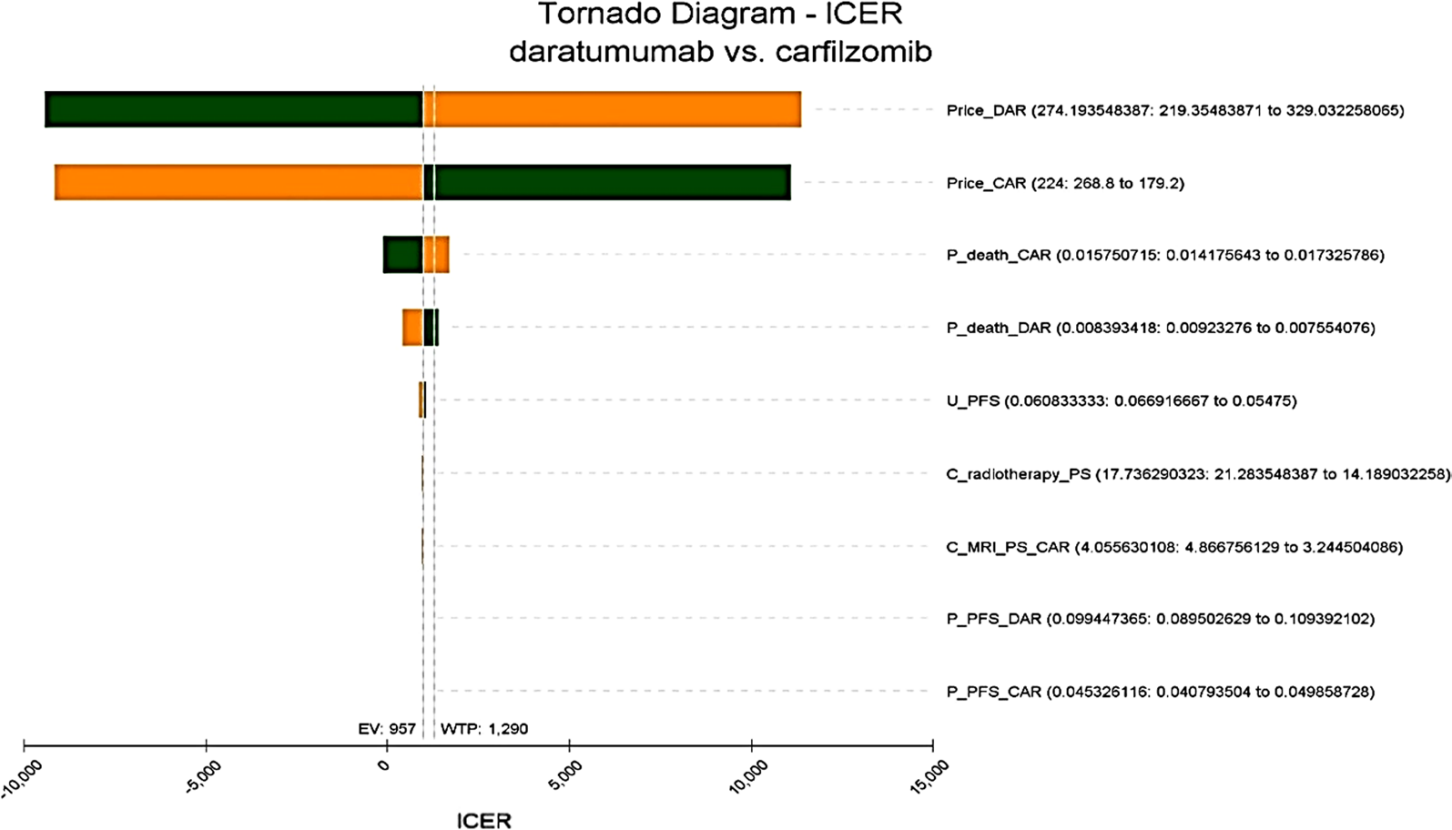
Tornado diagram. The green bar section representing the parameter range from the low uncertainty value to the base case, while the orange bar section represents the parameter range from the base case to the high uncertainty value. *DAR* Daratumumab, *CAR* Carfilzumib, *P* probability, *U* utility, *PFS* progression-free survival, *C* cost, *PS* progression state

One-way sensitivity analyses showed that the drug cost of Daratumumab and Carfilzomib were the most influential factors within the model. The results were robust based on the sensitivity analysis (Fig. 2).

**Fig. 3:**
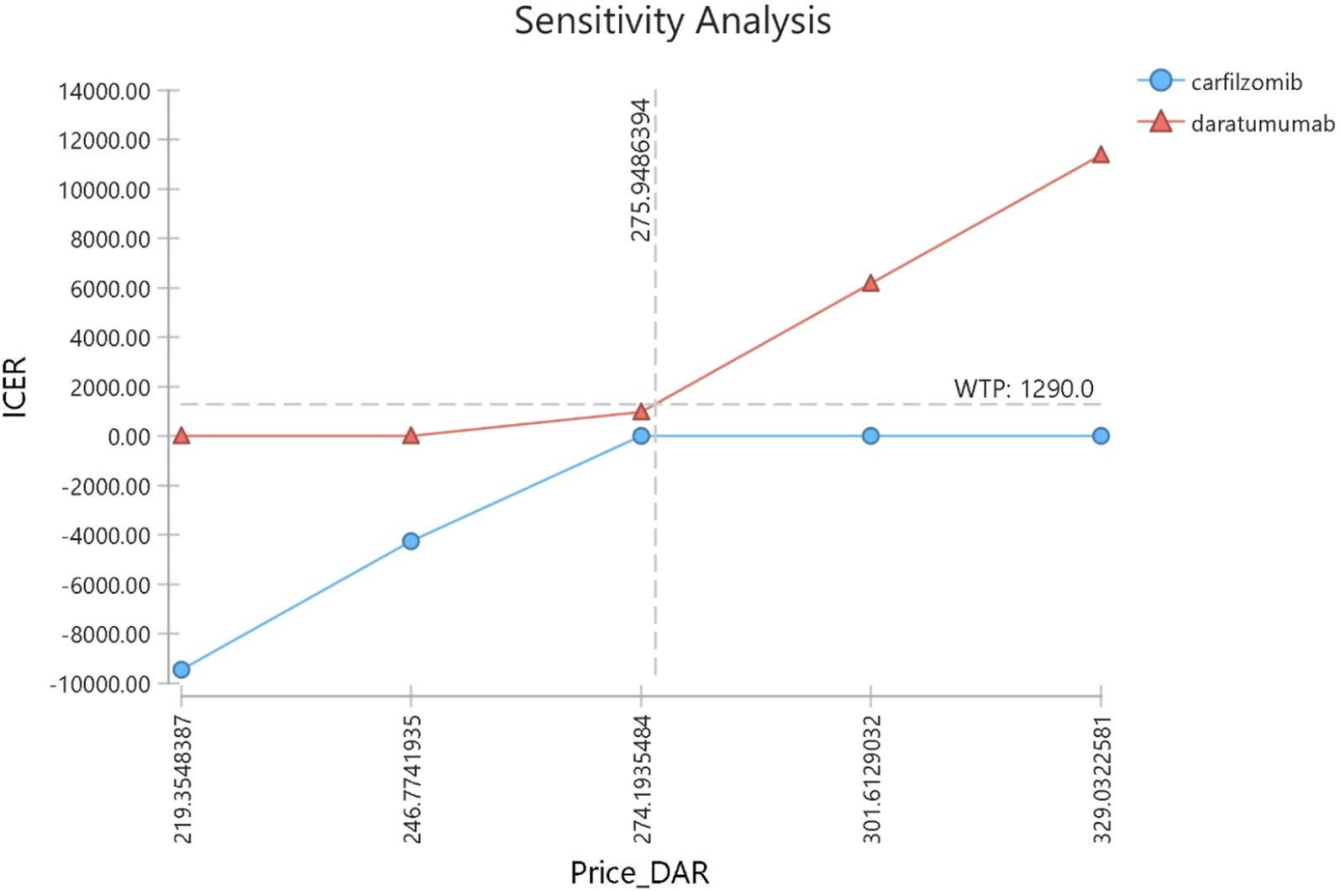
The results of one-way sensitivity analysis based on price changes of Dartumumab

The one-way sensitivity analysis in Fig. 2 shows that the DRd regimen would not be cost-effective if the price of Daratumumab was increased by $1. A $54 drop in drug prices would result in a negative ACER and the DRd regimen would become the dominant treatment regimen for MM (Fig. 3).

**Fig. 4:**
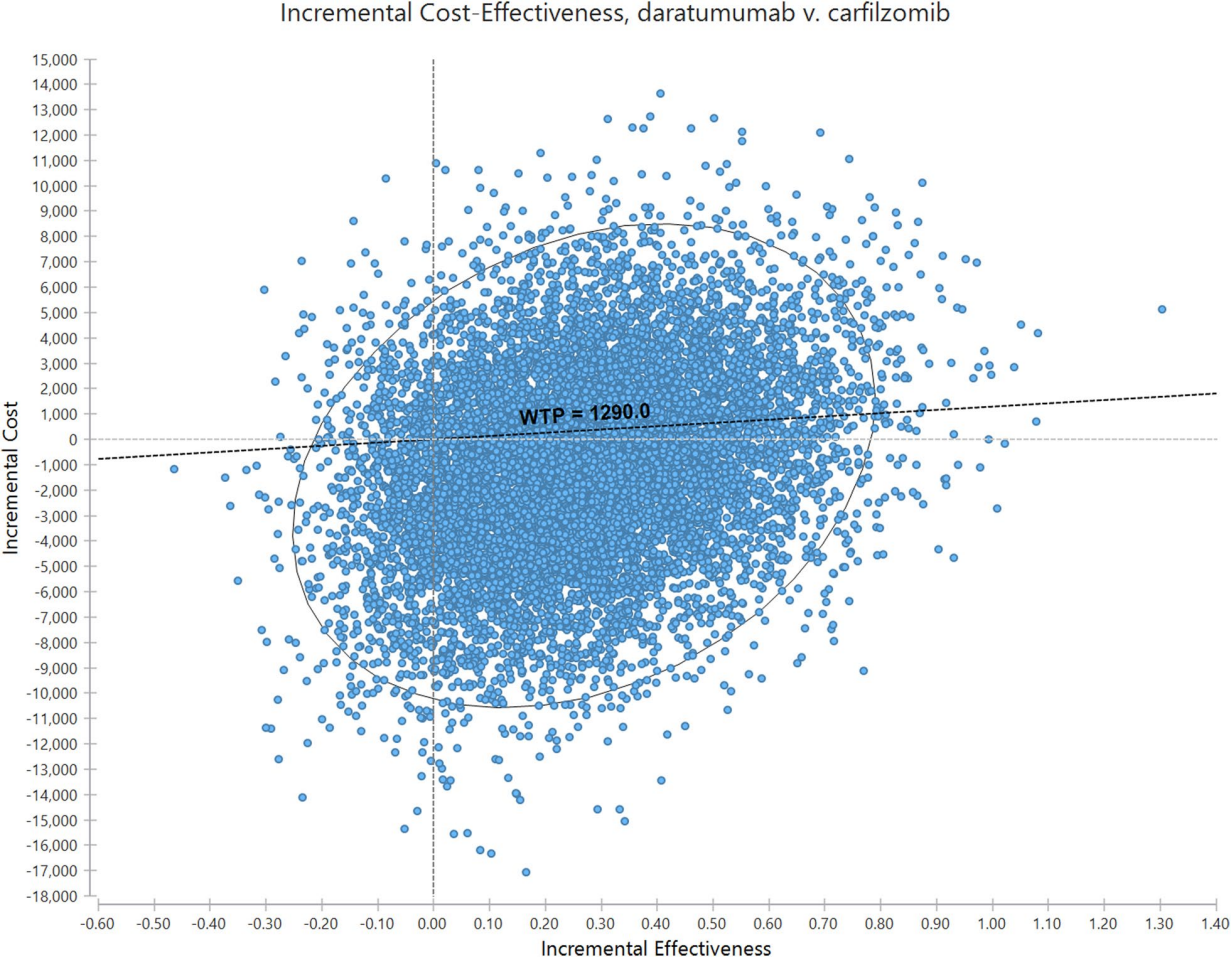
The results of Probabilistic Sensitivity Analysis (PSA)

**Fig. 5:**
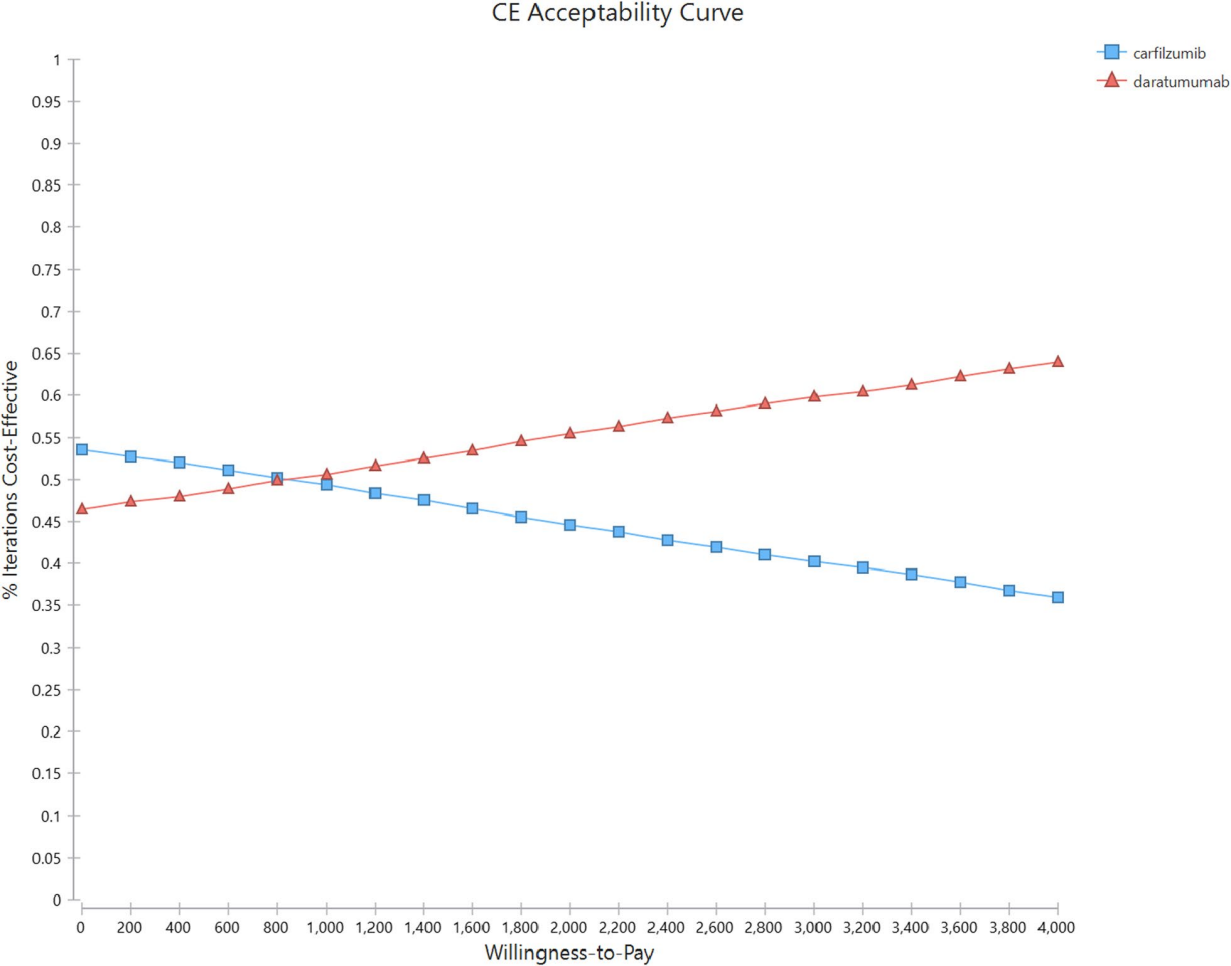
Cost-effectiveness acceptability curve based on ICUR between DRd and KRd. The horizontal axis displays the willingness-to-pay budgetary thresholds to gain one additional QALY when using DRd, and the vertical axis displays the percentage of 10,000 patients that fall within the available budget

The results of PAS are shown in Fig. 4. The scatter plot of incremental costs and QALYs shows that all simulations resulted in DRd being more effective and costlier than KRd. The probability of the DRd being cost-effective remained 55% under a WTP of $1290/QALY. In Fig. 5. Cost-effectiveness acceptability curve showing the probability that the new intervention is cost-effective as a function of the threshold. According to this figure the switch point where DRd became a cost-effective treatment corresponds to €810 per QALY. It also shows that at double ($2580) and triple ($3870/QALY) the initial threshold, the acceptable percentage of DRD is 60% and 65%, respectively.

### Budget impact analysis

The budget impact analysis estimated that once access to DRd regimen is established, the increase in spending on the health system is expected to be 3%, 7%, 12%, 16%, and 19%, respectively, by the fifth year. In other words, adding DRd regimen to the treatment basket of patients with RRMM, within a period of 5 years, increases the costs of the health system by 57%, which is equivalent to $6.170.582. The impact of the budget on the health financial burden of the government from 2022 to 2026 is presented in Table 3.

**Table 3:** Budget impact analysis results

Year	2022	2023	2024	2025	2026
Iran population	85,345,667	86,369,815	87,406,253	88,455,128	89,516,589
Number of patients of MM	4267	4318	4370	4423	4476
Number of patients of MM with adjusted death rate	3456	3498	3540	3582	3625
Total number of MM who deserve to receive DRd or KRd	691	700	708	716	725
KRD market share	0.95	0.9	0.85	0.8	0.75
Daratumumab market share	0.05	0.1	0.15	0.2	0.25
Scenario 1 (without DRd)	10,258,397	10,381,497	10,506,457	10,632,438	10,759,734
Scenario 2 (with DRd)	10,656,889	11,188,046	11,730,407	12,284,193	12,849,570
Financial impact	398,492	806,549	1,223,950	1,651,755	2,089,836
Financial impact%	3%	7%	12%	16%	19%

*DRd*, Daratumumab, Lenalidomide, dexamethasone, *KRd* Carfilzomib, Lenalidomide, dexamethasone

## Discussion

So far, a clinical trial study has not compared head-to-head KRd and DRd treatment regimens, but a network meta-analysis study has shown that the addition of Daratumumab to the dual-drug regimen of Lenalidomide and dexamethasone improves OS and PFS compared to the addition of Carfilzomib to the same regimen [24, 26].

In this study, the cost effectiveness of DRd compared to KRd in patients with MM who have received at least one previous treatment was investigated using the Markov model from the perspective of the Iranian payer. We used markov model for describing the clinical pathways of mutually exclusive health states through which a patient will progress during the disease, because a published study presented an application of the state transition modeling (STM), commonly applied as a Markov model, in relapsed multiple myeloma and showed that the STM accurately captures the underlying disease process over the modeled time period [32]. The results showed that compared to KRd, DRd resulted in an average of 2.34 additional life-years (5.86 vs 3.52), slightly more QALY (0.28) for slightly more cost ($264) and the obtained ICUR ($956) was below the WTP threshold ($1290). The results of one-way sensitivity analysis showed that the results are more sensitive to the price of Daratumumab. Also, the results of the Monte Carlo simulation of the cost effectiveness of Daratumumab in 55% of the simulations. Based on one-way sensitivity analysis, with a few increase in the price of Daratumumab, the treatment regimen will no longer be cost-effective. Also, based on the results of Monte Carlo simulation, Daratumumab will not be cost-effective in 45% of the performed simulations. The Iranian regulatory authorities suggest that the price of the new technology should be set at a level that in 70% of the simulations, it is cost effective. So, the price of Dartumumab should be reduced to $238 to be cost effective in 70% of the simulations. Calculations of the budget impact in this study showed that within 5 years, if Daratumumab is included in Iran's drug list and by acquiring 25% of the market share until the fifth year, this drug will lead to an increase of $6,192,000 in the costs of Iran's health system.

To our knowledge, this study is the first cost-effectiveness and budget-impact study conducted to compare DRd and KRd triple-drug regimens. Considering the difference in the price of these drugs and the difference in the duration of use of these drugs (Daratumumab is used for 25 cycles and Carfilzomib for 18 cycles), the increase in treatment costs with the use of Daratumumab seems reasonable, but the clinical advantages of Daratumumab over Carfilzomib make that the regimen containing Daratumumab is more cost effective than the regimen containing Carfilzomib in the treatment of MM.

To date, few studies have investigated the cost-effectiveness of DRd treatment regimen in combination with other MM treatments [33–35]. A 2020 study from Singapore [33] investigated the cost-effectiveness of DRd therapy compared with Rd in MM patients who had received at least one prior treatment. The ICER for the DRd treatment regimen was $576,247 per QALY. One-way sensitivity analysis showed that the results were highly sensitive to the cost of Daratumumab. The result of the study showed that DRd treatment regimen is not cost effective compared to Rd. In another study [34] conducted from a US healthcare perspective to investigate the cost-effectiveness of three treatment regimens DRd, VRd, and Rd in patients with MM ineligible for autologous stem cell transplantation, the results showed that Rd had the lowest overall cost at $329,867, followed by VRd at $385,434, and DRd at $626,900. Rd was estimated to contribute the least amount of QALY (1.24), followed by VRd with 1.35 and DRd with 1.52 QALY. At the WTP threshold of $150,000, DRd was more cost-effective than VRd and Rd, with ICERs of $1,396,318 and $1,060,832 per item, respectively. Another study [30] compared the cost-effectiveness of triple-drug therapies for patients with refractory or relapsed MM from a US payer perspective. The results show that at a WTP threshold of $150,000, the ICER for DRd compared with Rd was $1,369,062 per QALY, and that under no price reduction, the addition of Daratumumab to the Rd regimen would not be cost-effective.

In all studies, the DRd has been compared with the Rd, and the results show that adding Daratumumab to the common treatment regimen is not cost-effective despite the clinical benefits it creates. One of the most important factors influencing the study results in the one-way sensitivity analysis is the price of Daratumumab. One of the concerns of policy makers and clinicians in choosing triple-drug regimens for RRMM is the high cost of these treatments. When an innovative product such as Daratumumab is added to a common dual-drug regimen such as Rd, which itself contains an innovative product, it increases costs dramatically. Considering this issue, it also applies to Carfilzomib, so the small difference in the costs of these two drug regimens in our study can be justified.

In the near future, Daratumumab will go off-patent (2025) [36], and generic versions of this drug are expected to be available at lower prices. Of course, the effect of lower generic prices on the cost effectiveness analysis depends on the regimens being compared. However, future studies could provide more insight into the impact of generic drug prices on their cost-effectiveness. But the point that is noteworthy is that in clinical trials, triple-drug regimens have had more clinical benefit than dual-drug regimens and have been able to significantly increase PFS and OS [11, 16]. As a result, economic evaluation studies are a very suitable tool in the field of informing policymakers and clinicians about the value of Daratumumab-based triple-drug regimens and facilitating cost containment for the treatment of RRMM.

There are limitations in our study. First, due to the fact that so far, no clinical trial study has done a head-to-head comparison of DRd and KRd treatment regimens, we used the results of a network meta-analysis in this study. Although network meta-analysis is a powerful tool for indirect comparisons, in the absence of evidence from head-to-head studies, caution should be exercised in interpreting results. Second, due to the limitations of meta-analysis data, survival probabilities for patients treated with DRd and KRd were obtained by pooling data from the control groups of randomized controlled trials comparing drugs with placebo.

## Conclusion

In patients with RRMM who have received more than one prior therapy, adding Daratumumab to the current Rd treatment regimen increases disease burden and treatment cost compared with adding Carfilzomib to the same regimen. But considering that the calculated ICER is below the WTP threshold, DRd is a cost-effective treatment in patients with RRMM. Further research is needed in patients with RRMM who have received at least one prior treatment to compare the relative effectiveness of currently available treatments and their consequences on quality of life.

## Data Availability

The datasets used and/or analysed during the current study are available from the corresponding author on reasonable request.

## Abbreviations

PFS: Progression-free survival
DRd: Daratumumab, Lenalidomide and dexamethasone
KRd: Carfilzomib, Lenalidomide and dexamethasone
RRMM: Relapsed-refractory multiple myeloma
RCTs: Randomized clinical trials
ICER: Incremental cost effectiveness ratio
HRQoL: Health-related quality of life
MM: Multiple myeloma
OS: Overall survival
QALYs: Quality-adjusted life years
PD: Progressive disease
AEs: Adverse events
DSA: Deterministic sensitivity analysis
PSA: Probable sensitivity analysis
BIA: Budget impact analysis
NMB: Net monetary benefit
ACER: Average cost effectiveness ratio
WTP: Willingness to pay
